# Association Between Leucopenia, Transaminitis, Nonstructural Protein One Antigen (Ns1Ag) Level, and Thrombocytopenia in Adult Dengue Patients in a Tertiary Care Hospital: A Cross-Sectional Study

**DOI:** 10.7759/cureus.55622

**Published:** 2024-03-06

**Authors:** Karthick Ramalingam, Anand N N, Ramprasath Perumalsamy, Chejarla Likhitha, Aravind Raj

**Affiliations:** 1 Internal Medicine, Sree Balaji Medical College and Hospital, Chennai, IND

**Keywords:** leucopenia, ns1ag, alt, ast, thrombocytopenia

## Abstract

Background: The dengue virus is present throughout the tropics. Thrombocytopenia is one of the severe manifestations of the dengue virus. We studied the association of thrombocytopenia with serum transaminase level, leucopenia, and nonstructural protein one antigen (Ns1Ag) level.

Methods: Data were taken retrospectively from hospital records after obtaining ethical committee approval. In the study, we included 102 patients with acute febrile illness with clinical features suggestive of dengue fever (dengue Ns1Ag positive, dengue IgM positive, or both). We excluded patients with thrombocytopenia due to other causes. Patients' demographic, clinical, and laboratory parameters were collected. We also noted episodes of bleeding or the need for a platelet transfusion. We did a statistical analysis to find out the correlation between age, sex, leucopenia, transaminitis, Ns1Ag level, and thrombocytopenia and its severity.

Results: Multiple regression analysis was used to find thrombocytopenia predictors among aspartate transaminase (AST), alanine transaminase (ALT), Ns1Ag level, and leucopenia. AST and ALT correlated inversely with thrombocytopenia, with p-values of 0.012 and 0.027, respectively. Ns1Ag and leucopenia were not associated with thrombocytopenia, with p-values of 0.802 and 0.532, respectively (p-values significant at 0.01<= p<=0.05).

Conclusion: Serum AST and ALT levels correlate with thrombocytopenia in dengue fever.

## Introduction

Dengue fever is caused by dengue virus belonging to the Flavivirus family. Four serotypes (DENV 1 to 4) are present in the dengue virus, but their clinical manifestations are indistinguishable. Clinical manifestations vary from mild febrile illness to severe dengue with thrombocytopenia, bleeding tendency, and shock. Dengue virus is transmitted by female Aedes mosquito [1].

Dengue virus infection is widespread throughout the tropics and is considered to increase as urbanization and globalization increase [2]. The incidence of dengue has risen more than 30 times in recent years and is regarded as one of the neglected diseases of the tropics that cause 21,000 deaths per year worldwide [3,4]. India accounts for 34% of global dengue burden. In 2009, WHO classified dengue with or without warning signs or severe dengue [5]. Warning signs include abdominal pain or tenderness, persistent vomiting, clinical fluid accumulation, mucosal bleeding, lethargy or restlessness, hepatomegaly of more than 2 cm, and an increased hematocrit level that is accompanied by a decrease in platelet count. The main pathological changes associated with dengue fever are plasma leakage and abnormal hemostasis. Dengue virus, when it infects the liver, can cause apoptosis of the hepatocytes. One of the clinical parameters associated with severe dengue is thrombocytopenia which occurs due to peripheral destruction of platelets and bone marrow suppression.

The study aims to find an association between leucopenia, transaminitis, nonstructural protein one antigen (Ns1Ag) level, and thrombocytopenia in adult dengue patients. The objective is to identify risk factors associated with thrombocytopenia so that better monitoring can be done in selected patients in resource-limited settings.

## Materials and methods

A cross-sectional study was done retrospectively from the medical records of a teaching hospital in southern India after obtaining ethical committees' approval (002/SBMC/IHEC/2021/1555). The period of study was April 2021 to June 2022. Patients with acute febrile illness with clinical features suggestive of dengue fever with Dengue Ns1Ag positive or Anti dengue IgM positive or both were included in the study. Patients having portal hypertension, idiopathic thrombocytopenic purpura, and hematological malignancy were excluded from the study. Clinical features of dengue were based on WHO 2009 guidelines, that is, the patient has recently traveled to or lives in an endemic area and has a fever and two of the following: nausea or vomiting, rash, aches and pains, positive tourniquet test, leucopenia, or any warning sign. Dengue serology was done using the ELISA method.

Detection of Dengue Ns1Ag antigen was done by microwell ELISA test. Anti-dengue IgM antibodies were detected using enzyme immunoassay based on MAC Capture ELISA. Anti-dengue IgG antibodies were detected using enzyme immunoassay based on GAC capture ELISA. If Ns1Ag or anti-dengue virus IgM was found in more than 11 units, the patient was considered to have dengue fever. Patients with Ns1Ag or Anti-Dengue virus IgM less than nine units were considered not to have dengue fever. Patients with values between nine and 11 units were supposed to be equivocal for dengue. Anti-dengue virus IgM antibodies are the most widely used test to diagnose dengue [6]. Patients with thrombocytopenia due to other causes like portal hypertension, immune thrombocytopenic purpura, liver disease, hematological malignancy, and autoimmune causes for thrombocytopenia were excluded.

We estimated sample size by using nMaster software Version 2.0 by applying the following details in the formula n= Z21-α/2p(1-p)/d2, where p is expected proportion (0.4031) [7], d is absolute precision (10%), 1-α/2 is desired confidence interval (95%). Based on the above parameter with an alpha of 0.05 (2-sided) and precision level of 10%, the estimated sample size uses the sample size formula for a single proportion. The above parameter and formula gave us a sample size of 92 subjects; considering the data loss, ten more samples were added, and 102 samples were taken in the present study.

The patient's symptoms were noted, such as fever, myalgia, joint pain, headache, rashes, and bleeding manifestations. The patient's comorbid illnesses like diabetes, hypertension, coronary artery disease, and chronic kidney disease were recorded, along with drugs taken by the patient. Patient's clinical details like hydration status, purpura or petechiae in the skin, blood pressure, and pulse rate were noted. Baseline complete hemogram, blood sugar, renal function test, and liver function test were collected. All these data were collected from physician's notes and medical records.

The patient's leukocyte count was done using the automated laser flow cytometry method. The patient's platelet count was done using the computerized sheath fluid impedance method. Patient's alanine transaminase (ALT) and aspartate transaminase (AST) were done using the UV Kinetic IFCC method. The patient's quantitative Ns1Ag level was noted. The patient's serial platelet counts were collected. Episodes of bleeding, including new petechiae or purpura, melena, gum bleeding, hematemesis, or need for platelet transfusion, were also noted.

The collected data were analyzed with IBM SPSS Statistics for Windows, Version 23.0 (IBM Corp., Armonk, NY). To describe the data descriptive statistics frequency analysis, and percentage analysis were used for categorical variables and the mean and SD were used for continuous variables. To find the platelet predictors the Multiple regression analysis with enter method was used. To assess the relationship between the variables Pearson's correlation was used. In both the above statistical tools the probability value of 0.05 is considered a significant level. Analysis was done to find out the correlation between leukopenia (WBC count less than 4,000 cells/dL), transaminitis (ALT or AST or both more than 40 U/L), Ns1Ag level (units), and thrombocytopenia and its severity (less than 1.5 lakh/dL).

## Results

After reviewing the medical records, 102 patients were included in the study. 53.9% of them were female, and 46.1% of patients were male. Headache (65.7%) was the most common symptom. Low back pain (48%), abdomen pain (25.5%), joint pain (46.1%), and diarrhea (14.7%) were the other common symptoms of the patients included in the study. Bleeding episodes were present in 7.8% of patients, though none had any significant bleeding. Platelet transfusions were given to 10 patients (Table 1).

**Table 1:** Demographics and clinical features (n=102)

Baseline demographic and clinical presentation of study population (N = 102)	
Age, years median (range)	26.98 (21.25 – 30) years
Male n,(%)	47(46.1%)
Fever Duration in days	3.94 ± 1.48 days
Headache n,(%)	67 (65.7%)
Low Back Ache n,(%)	49 (48%)
Abdominal Pain n,(%)	26 (25.5%)
Joint pain n,(%)	47 (46.1%)
Diarrohea n,(%)	15 (14.7%)
Bleeding manifestations n,(%)	8 (7.8%)

Leucopenia was present in 64.70% of patients. Platelets less than 50,000 were seen in 41.176% of patients. AST (SGOT) was elevated (>40 IU/mL) in 70.5% of patients, and ALT (SGPT) was elevated (>40 IU/mL) in 60.78% of patients. Eighty-seven patients had Ns1Ag positive (Table 2).

**Table 2:** Descriptive statistics of laboratory parameters of study population WBC - White Blood Cells

	Mean	Standard Deviation	Minimum	Maximum	Reference Range
Body Temperature(Farenheit)	101.17	1.23	98.50	105	97.7-99.5
Maximum Systolic Blood Pressure(mmHg)	118.03	10.53	90.0	150	139
Maximum Diastolic Blood Pressure(mmHg)	78.03	8.56	60.0	110	89
Minimum Systolic Blood Pressure(mmHg)	103.52	10.77	70	120	90
Minimum Diastolic Blood Pressure(mmHg)	66.66	7.75	30	80	60
Hemoglobin(g/dl)	13.18	1.94	9.20	19	12-15
Admission Hematocrit (fl)	39.34	5.13	25.10	54.6	36-46
Discharge Hematocrit(fl)	39.09	5.03	28.70	52.8	36-46
Maximum Hematocrit(fl)	44.66	35.61	28.80	397	36-46
WBC count(*10^6^ cell/L)	3808.58	2303.84	336.0	18900	4000- 10000
Admission platelet count(cell/mm^3^)	123131.37	60799.82	10000	302000	150000 -410000
Lowest platelet Count(cell/mm^3^)	72156.86	51339.62	6000	244000	150000 -410000
Discharge platelet count(cell/mm^3^)	145901.96	128147.30	42000	1300000	150000 -410000
T.Bilirubin(mg/dl)	0.54	0.33	0.1	2.2	0.3-1.1
Aspartate transaminase(IU/L)	118.40	164.11	15	934	<40
Alanine transaminase(IU/L)	92.04	116.32	10	761	<40
Alkaline phosphatase(IU/L)	75.63	32.31	30	187	53-128
Total Protein(g/dl)	6.66	0.61	4.1	8.3	6-7.8
Albumin(g/dl)	3.89	0.42	2.8	5.3	3.35-5.2
Globulin(g/dl)	2.80	0.50	1.1	3.9	2.5-3.5
Non structural Antigen(NS1Ag)Units	70.90	42.87	0.540	189.22	0-11
Immunoglobulin M(IgM)Units	21.09	25.85	0.1	132.18	0-11
Immunoglobulin G(IgG)Units	14.87	22.92	0.1	112.3	0-11

Univariate analysis was used to find thrombocytopenia predictors among AST, ALT, Ns1AG level, and leucopenia. AST and ALT correlated inversely with thrombocytopenia, with a p-value of 0.012 and 0.027, respectively. Ns1Ag and leucopenia were not associated with thrombocytopenia with p-values of 0.802 and 0.532, respectively (p-value significant at 0.01<= p<=0.05). The multiple regression analysis to find the predictors of low platelets shows that no factors were statistically significant predictors (Tables 3, 4).

**Table 3:** Risk factor analysis of thrombocytopenia among the study population Pearson's Correlation * Correlation is significant at the 0.05 level (2-tailed). ** Correlation is significant at the 0.01 level (2-tailed). AST - Aspartate transaminase, ALT - Alanine transaminase, WBC - White Blood Cell, Ns1Ag - Nonstructural one antigen

		Lowest platelet count	AST	ALT	WBC count	Ns1Ag
Lowest platelet count	r-value	1	-0.249*	-0.218*	0.063	-0.025
	P-value		0.012	0.027	0.532	0.802
	N	102	102	102	102	102

**Table 4:** Multiple regression analysis for risk factors of thrombocytopenia Dependent Variable: Lowest platelet Count AST - Aspartate transaminase, ALT - Alanine transaminase, WBC - White Blood Cell, Ns1Ag - Nonstructural one antigen

Model	Unstandardized Coefficients		Standardized Coefficients	t	Sig.	95.0% Confidence Interval for B	
	B	STD. ERROR	Beta			Lower Bound	Upper Bound
Constant	73403.508	14038.787		5.229	0.000	4,5540.402	143,550.17
WBC count	1.294	2.195	0.059	0.589	0.557	-3.063	5.650
AST	-89.714	76.455	-0.290	-1.173	0.244	-241.457	62.029
ALT	19.909	107.929	0.046	0.184	0.854	-194.299	234.117
Ns1Ag	-4.424	117.999	-0.004	-0.037	0.970	-238.618	229.771

## Discussion

Dengue infection has a case fatality rate of around 2.5% to 5.4% [8,9]. With better monitoring and early treatment, the mortality rate can be decreased [10]. During monitoring, vital signs, platelet count, and hematocrit are measured at least two to three times a day. According to WHO, thrombocytopenia is one of the indicators of the clinical severity of the disease. Dengue patients with thrombocytopenia have seven times more chance of having leucopenia than those who don't have thrombocytopenia. Monitoring patients frequently is difficult in resource-limited settings. Therefore, the correlation between thrombocytopenia, ALT, AST, WBC count, and Ns1AG will help us to triage patients better.

In our study, among the four factors studied in dengue fever, only AST and ALT correlated inversely with thrombocytopenia in dengue. Ns1Ag level and leucopenia did not correlate with thrombocytopenia. Dengue virus can produce decreased production of all three cell lineages by bone marrow depression early in the illness. Once the febrile phase is over, WBC count and RBC count will improve, leaving a residual decrease in platelets that may result in bleeding tendencies [11,12]. Dengue virus can cause thrombocytopenia due to platelet destruction and bone marrow suppression. In our study, thrombocytopenia of less than 50,000 was seen in 41.18% of patients compared with previous studies by Agrawal et al., where platelets less than 50,000 were seen in 58.9% of patients [13].

Hepatomegaly, direct cytopathic effect of dengue virus, immune-mediated hepatitis, cytokine storm, and apoptosis of hepatocytes can occur in dengue fever, causing a rise in ALT and AST. Transaminase elevation is usually only mild to moderate in dengue patients. The conjugation function of the liver is generally preserved, so jaundice is not frequently seen in dengue patients [14,15]. In our study, elevated AST and ALT were seen in 70.5% and 60.78%, respectively. In a study by Rao et al. in dengue patients, AST and ALT elevation was seen in 85% and 84% of patients, respectively [16]. In our study, AST and ALT levels correlated with thrombocytopenia in univariate analysis, but in multiple regression analysis, none were independent risk factors associated with thrombocytopenia. In another study, ALT level correlated with dengue severity [17]. As with previous studies, jaundice was not seen in our patients.

In our study, though leucopenia was seen in 64.70% of patients, a significant correlation was not established between leucopenia and thrombocytopenia. In a study by Chacko and Subramanian leucopenia was associated with severe dengue [17]. Ns1 Ag takes part in the multiplication of the dengue virus. Secreted Ns1Ag antigen can be detected as early as the first day of dengue fever, and it can be quantified. Blood levels of this antigen correlate with peak viremia. In some studies, blood level is associated with the severity of dengue [18]. It can be identified in most fluids of the body [19]. In our study, Ns1Ag level did not correlate with thrombocytopenia severity. In our study, Ns1Ag level was seen only once. If serial Ns1Ag levels were done, we may be able to see whether peak Ns1Ag level correlates with thrombocytopenia.

The limitations of our study were the small sample size, retrospective design, and fact that it was done in a single center. In a prospective design, serial Ns1Ag level can be done, and we may be able to find whether peak level of Ns1Ag levels correlate with thrombocytopenia.

## Conclusions

Serum aspartate and alanine transaminase levels correlate with thrombocytopenia in patients with dengue fever. In patients with raised transaminase levels close monitoring for thrombocytopenia and bleeding manifestations should be done. A more extensive study is needed to confirm the findings of this study.
